# *Lignosus rhinocerus* (Cooke) Ryvarden: A Medicinal Mushroom That Stimulates Neurite Outgrowth in PC-12 Cells

**DOI:** 10.1155/2012/320308

**Published:** 2011-12-06

**Authors:** Lee-Fang Eik, Murali Naidu, Pamela David, Kah-Hui Wong, Yee-Shin Tan, Vikineswary Sabaratnam

**Affiliations:** ^1^Mushroom Research Centre, University of Malaya, 50603 Kuala Lumpur, Malaysia; ^2^Institute of Biological Sciences, Faculty of Science Building, University of Malaya, 50603 Kuala Lumpur, Malaysia; ^3^Department of Anatomy, Faculty of Medicine, University of Malaya, 50603 Kuala Lumpur, Malaysia

## Abstract

A national treasure mushroom, *Lignosus rhinocerus*, has been used to treat variety of ailments by local and indigenous communities in Malaysia. The aim of this study was to investigate the potential of the most valuable part of *L. rhinocerus*, the sclerotium, on neurite outgrowth activity by using PC-12Adh cell line. Differentiated cells with one thin extension at least double the length of the cell diameter were scored positive. Our results showed that aqueous sclerotium *L. rhinocerus* extract induced neurite outgrowths of 24.4% and 42.1% at 20 **μ**g/mL (w/v) of aqueous extract alone and a combination of 20 **μ**g/mL (w/v) aqueous extract and 30 ng/mL (w/v) of NGF, respectively. Combination of NGF and sclerotium extract had additive effects and enhanced neurite outgrowth. Neuronal differentiation was demonstrated by indirect immunofluorescence of neurofilament protein. Aqueous sclerotium extract contained neuroactive compounds that stimulated neurite outgrowth *in vitro*. To our knowledge this is the first report on neurite-stimulating activities of *L. rhinocerus*.

## 1. Introduction

Formation of central nervous system (CNS) network requires active axonal elongation and systematic explorations activity of environment by growing of axons in order to be directed to their correct target [1]. Neuronal cells are able to sense the surrounding and form branches in response to molecular information from extracellular milieu that instruct the maturation process and induce neurite regeneration in pathological situations such as trauma or degenerative diseases [1]. Neurite outgrowth in cultured neurons is considered as an indication of neuroregenerative potential [2].

Extension and remodeling of neurites play essential roles in development and neuronal plasticity regulated by neuroserpin [3]. Cultured rat pheochromocytoma PC-12 cells have been used extensively as an *in vitro* model system for investigation of neuronal differentiation. Epidermal growth factor (EGF) and nerve growth factor (NGF) both activate extracellular signal-regulated kinase (ERK) and p38 mitogen-activated protein kinase (MAPK) [4]. But PC-12 cells only respond to neurotrophin NGF and differentiate into sympathetic neuron phenotype and extending axon-like processes called neurites [3]. This serves as an excellent model to study the effects of molecules both synthetic and natural that will stimulate the outgrowth of neurites.

Of the many diseases that threaten humans, neurodegenerative diseases can be very traumatic as one ages. Neurohealth is the concern for the predicted silver tsunami to hit humans—the aging tsunami is projected to be 80–90 million of 65-plus population in 2050 [5]. Why the concern? Alzheimer and neurogenerative diseases are high on the list of chronic diseases of the aged. Alzheimer's disease is primarily a disorder of aging with loss of cognitive function. This disease is characterized biologically by the death of neurons in the forebrain, hippocampus, and cerebral cortex accompanied by the presence of amyloid deposition.

Many of the drugs in the market only delay further deterioration and do not reverse the damage done to cognitive functions. The search is now for small molecules that can cross the brain-blood and induce the production of nerve growth factor (NGF), a family of proteins responsible for maintenance, survival, and regeneration of neurons during adult life. It has been shown that NGF absence causes an Alzheimer's like symptoms in the adult brain of mice. We may have to turn to nature to prevent or reduce the severity of nerve-related diseases as we age. Prevention will be definitely better than cure. Many plants and spices such as turmeric are said to help. For example, in India, it is noted that Alzheimer among the older generation is not in alarming numbers. The regular consumption of spices including turmeric and pepper may be the reason and currently this is actively been studied. Currently mushrooms are also being investigated as sources of NGF stimulators.

Mushroom, a macrofungus that has either culinary or medicinal properties, has a visible fruiting body. Culinary mushrooms have been known as famous appetizing and nutritious food all over the world. Historically, medicinal mushrooms used by different tribes are best documented in the eastern world.

In Malaysia, several macrofungi are used by the Malays, Chineses, and indigenous communities for treatment of variety ailments. *Lignosus rhinocerus* has been singled out as one of the most potent mushrooms for medicinal purposes dating to the 1700s by Tuan Haji Mat Yusop, a Malay in Pahang [6, 7]. *Lignosus rhinocerus* is a unique “National Treasure” that can only be found in a small geographic region encompassing South China, Thailand, Malaysia, Indonesia, Philippines, Papua New Guinea, New Zealand, and Australia [6]. *Lignosus rhinocerus* (Cooke) Ryvarden is also known as “cendawan susu rimau” in Malay language or Tiger's Milk mushroom in English. The medicinal properties recorded by ethno-mycological surveys are yet to be validated scientifically.

*Lignosus rhinocerus* has more than 15 medicinal uses. Different natives use it as an antipruritic, antipyretic, general tonic, starve off hunger, cancer, food poisoning, swollen breasts [7], fever, cough, asthma, wound healing, and others [6]. Although this mushroom has been recorded to have a number medicinal properties, usage is limited due to unavailability of the mushroom. The underground fungus tuber or sclerotium is the part with medicinal value and it can only be noticed when the fruiting body sprouts out from the ground when nature calls. Recently, efforts made to cultivate this medicinal mushroom have been successful [8] and the mushrooms are now available for scientific studies.

The aim of the study was to assess the presence of active agents in aqueous extract of *L. rhinocerus* sclerotium that can stimulate neurite outgrowth in PC-12 and the enhancement of neurite outgrowth with the combination of NGF and aqueous extract in PC-12 cells. Quantitative morphological methods and neurofilament formation were used to study the differentiation effects on neurite outgrowth activities in PC-12 cell line. To our knowledge, this is the first report on the application of sclerotium of *L. rhinocerus* in neurite outgrowth stimulation activity.

## 2. Methods

### 2.1. Preparation of Aqueous Extract Sclerotium

*Lignosus rhinocerus* sclerotium freeze-dried powder was from Dr. Tan Chon Seng of Malaysian Agricultural Research and Development Institute (MARDI). The aqueous extraction method was modified by Wong et al. [9]. Sclerotium powder was weighed; distilled water at a ratio of 1 : 20 (w/v) was added and left for 24 hr at 27 ± 2°C at 150 rpm. Then the mixture was double boiled for 30 min, cooled, and filtered through Whatman filter paper No. 4. The aqueous extract was then freeze-dried at −50 ± 2°C for 48 hr and stored in airtight bottles at −40°C prior to assay.

### 2.2. *In Vitro* Cell Culture

PC-12 cell line, derived from transplantable rat pheochromocytoma, has been widely used as neuronal model because it proliferates in growth medium, and stop proliferating and differentiate into neuron-like cells as they respond to NGF [10]. The cell line has been used to study mechanisms of action of neurotoxicants and potential of chemical to alter neurite differentiation [11]. PC-12 cells are grown as floating clusters and few scattered lightly attached cells; therefore adherent variant (PC-12Adh) was designed to improve cell attachment on flask.

PC-12Adh was chosen for this study because it is NGF-dependent and does not synthesize epinephrine. PC-12 cell line was purchased from American Type Culture Collection (Cat #: CRL-1721.1) and only early passage cells were used in this study. The cells were cultured in ATCC-formulated F-12K Medium (Kaighn's Modification of Ham's F-12 Medium) supplemented with 2.5% (v/v) fetal bovine serum and 15% (v/v) horse serum with final pH 6.8–7.2 and incubated in 5% CO_2_ humidified incubator at 37 ± 2°C [12]. The cells were subcultured every 2 to 3 days as needed. PC-12 cells tend to form clusters and clumps. For subculturing, cells were detached from culture flask by scraping and this forceful aspiration was employed to break up cell clusters [13]. For storage, the cells were frozen in complete F-12K medium with 5% (v/v) dimethylsulfoxide (DMSO) [10].

### 2.3. Neurite Stimulation Activity Assay

Cells were cultured for 2 to 3 days until 60–70% confluent prior to assay. They were plated into 12-well plates at cell density of 5 × 10^4^ cells per well in complete F-12K medium with concentrations 1 to 500 *μ*g/mL (w/v) of sclerotium aqueous extract. Concentrations of NGF-7S from murine submaxillary gland (Sigma, St. Louis, MO, USA) ranging from 10 ng/mL (w/v) to 100 ng/mL (w/v) were tested to examine the optimum concentration for neurite stimulation activity. The optimum concentration was used as positive control for the following assays. Cells in complete F-12K medium without treatment served as negative control. Freeze-dried aqueous extracts were diluted to various concentration with sterilized distilled water. After the preliminary test, optimum concentration of the sclerotium aqueous extract in combination with NGF ranging from 10 ng/mL (w/v) to 50 ng/mL (w/v) was tested to evaluate synergistic interaction, if any, between sclerotium aqueous extract and NGF. Assay plates were incubated at 37 ± 2°C in a 5% CO_2_-humidified incubator. Differentiation activity of cells in terms of neurite outgrowth and branching was observed after 48 hr of incubation at 37 ± 2°C in 5% CO_2_-humidified incubator.

### 2.4. Neurites Scoring

A cell was scored positive for bearing neurites if it has a thin neurite extension that double the length of the cell body diameter. Morphology of the cells is polygonal. Cells with irregular patterns such as sheet-like spreading cells, rare radially oriented possess, and apparently arising by “shrink-age” were excluded [9, 12] and cell clumps with more than five cells in a clump were excluded [9]. Ten fields per well were randomly examined and photographed under Nikon Eclipse TS100 with 10 × 10.25 Nikon objective and captured with Nikon DS-Fi1 camera and Nikon's Imaging Software, NIS-Elements. The percentage of neurite-bearing cells were quantified by scoring total number of neurite-bearing cells and total number of viable cells in 10 microscopic fields with average of 200 to 300 cells per well.

### 2.5. Immunofluorescence Staining of Neurofilaments

Immunofluorescence staining of neurofilament protein was carried out according to the method of Schimmelpfeng et al. [18]. Cells were seeded onto 6-well plates where each well contained two sterilized coverslips and incubated for 2 days at 37 ± 2°C in a 5% CO_2_-humidified incubator. On the day of experiment cells were 20% to 50% confluent and were fixed with 4% parafamaldehyde at room temperature for 20 min. The cells were then incubated with primary antibody, anti-neurofilament 200 antibody produced in rabbit (1 : 80 dilution in blocking buffer, Sigma, St. Louis, MO, USA) at room temperature for 1 hr, washed with washing buffer, and followed by further reaction with the secondary antibody, Fluorophore-conjugated secondary antibody, Anti-Rabbit IgG-Fluorescein isothiocyanate (FITC) antibody produced in sheep (1 : 80 dilution in blocking buffer, Sigma, St. Louis, MO, USA) at room temperature for 1 to 2 hr in dark. After the same washing procedure, cells were mounted onto slides with aqueous mounting medium, with 4′-6-Diamidino-2-phenylindole (DAPI). DAPI is used to stain nuclei. Finally, slides were observed under Nikon Eclipse 80i microscope under fluorescence illumination using FITC and DAPI filters and images were captured with Nikon's Imaging Software, NIS-Elements.

### 2.6. Statistical Analysis

Statistical analysis was performed using the computing environment R (R Development Core Team, 2011) (Vienna, Austria). Permutation test was used to examine the statistical significance of the differences [15]. Confidence intervals provide information on the direction and strength of the effect of the treatment [16, 17]. Therefore, confidence intervals (CIs) for all data were set to 95% (*P* < 0.001) [16].

## 3. Results and Discussion

### 3.1. Neurite Stimulation Activity Assay

**Figure 1:**
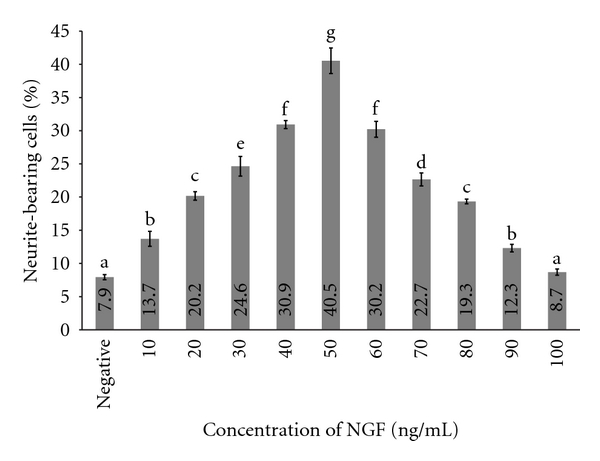
Percentage of neurite-bearing cells in the cell line PC-12 in response to treatment with various concentrations of NGF (ng/mL). Neurite growth (vertical axis) was quantified as the percentage of cells bearing axodendritic processes longer than two times cell diameters in length. Negative control consists of PC-12 cells in medium only. Data are expressed as means ± standard deviation (*n* = 3). Means with different alphabets show significant difference (Duncan's Multiple Range Test (DMRT)) (permuted-*P* < 0.001).

Neurite outgrowth stimulatory of NGF and aqueous extract of *L. rhinocerus* was observed after 48 hr of incubation. The uniqueness of PC-12 cell line is that it responds to NGF with a drastic change in its phenotype and cause proliferation, extension of neurite [11].  Figure 1 shows the effect of various concentrations of NGF on neurite stimulation activity of PC-12 cell line.

NGF concentration plot (Figure 1) determined the optimum concentration needed to induce maximal neurite outgrowth of PC-12. Percentage of neurite-bearing cell increased when exposed to NGF ranging from negative control which is medium alone to 50 ng/mL (w/v) and decreased when exposed to 60 ng/mL to 100 ng/mL (w/v) of NGF. When the cells were exposed to 50 ng/mL (w/v) of NGF, it showed a maximal effect with an increase of 33% (95% CI: 31–34) or almost five times increase of percentage of neurite-bearing cells compared with medium alone (negative control). Therefore, 50 ng/mL (w/v) of NGF was used as positive control for the subsequent assays.

**Figure 2:**
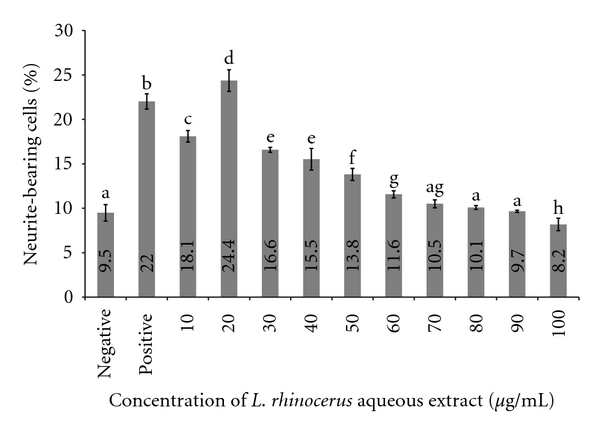
Percentage of neurite-bearing cells in the cell line PC-12 incubated with various concentrations of *L. rhinocerus* aqueous extract (*μ*g/mL). Neurite growth (vertical axis) was quantified as the percentage of cells bearing axodendritic processes longer than two times of cell diameters in length. Negative control consists of PC-12 cells in medium only whereas in positive control the cells were treated with 50 ng/mL (w/w) NGF. Data are expressed as means ± standard deviation (*n* = 3). Means with different alphabets show significant difference (*P* < 0.01; Duncan's Multiple Range Test (DMRT)) (permuted-*P* < 0.001).

Aqueous extract of *Lignosus rhinocerus *showed visible neurite outgrowth activity of PC-12 after 48 hr of incubation (Figure 2). Maximal stimulation was recorded at 24.4% at 20 *μ*g/mL (w/v) with an increase of 15% (95% CI: 13–16) compared to negative control and 2.5% (95% CI: 1–4) higher compared to positive control. Increasing the concentration showed minimal or even negative effect on the number of neurite-bearing cells. There were no significant differences between negative control and addition of extract at concentration of 80 *μ*g/mL (w/v) and 90 *μ*g/mL (w/v). At 100 *μ*g/mL (w/v) when compared to a negative control, a reduction of 1.3% (95% CI: (−2)–(−0.3)) of percentage of neurite-bearing cells was recorded.

**Figure 3:**
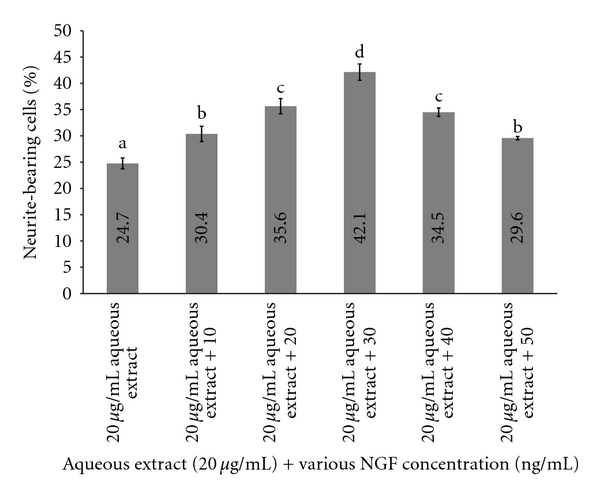
Percentage of neurite-bearing cells in the cell line PC-12 treated with combination of 20 *μ*g/mL (w/v) of aqueous extract and various concentrations NGF (ng/mL). Neurite growth (vertical axis) was quantified as the percentage of cells bearing axodendritic processes longer than two times of cell diameters in length. Data are expressed as means ± standard deviation (*n* = 3). Means with different alphabets show significant difference (*P* < 0.01; Duncan's Multiple Range Test (DMRT)) (permuted-*P* < 0.001).

From the above results (Figure 2), 20 *μ*g/mL (w/v) of aqueous extract of *L. rhinocerus *was selected as the optimum concentration for neurite outgrowth activity. The percentage of cells treated with various concentrations of NGF and optimum concentration of aqueous extract at 20 *μ*g/mL (w/v) showed increase in number of neurite-bearing cells compared to cells treated with optimum concentration of aqueous extract alone (Figure 3). This showed that combination of NGF and aqueous extract could enhance stimulation of neurite outgrowth. Optimum concentration of aqueous extract added with 30 ng/mL of NGF showed the best stimulation of neurite outgrowth (42.12%) and a 17% (95% CI: 16–19) increase in neurite-bearing cells compared to aqueous extract alone. Our data showed that the combination of aqueous extract and a lower NGF concentration was able to enhance neurite outgrowth activity comparable to the stimulation activity of a higher concentration of NGF at 50 ng/mL (w/v).

### 3.2. Immunofluorescence Staining of Neurofilaments

**Figure 4:**
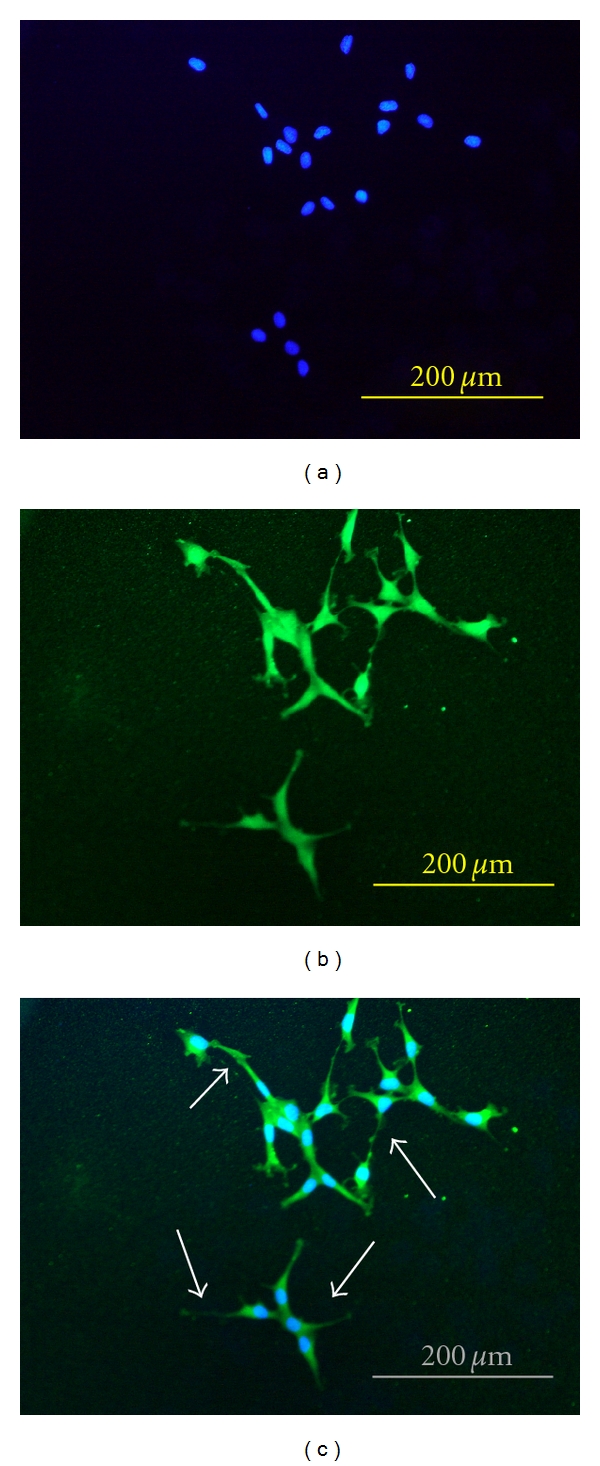
Neurofilament stain on PC-12 cells showing (a) DAPI staining for nuclei; blue, (b) anti-neurofilament 200 kD labeled with FITC staining for neuronalcells; green, and (c) the merged image; nuclei in blue and neuronal cells in green. Arrows indicate neurite outgrowth.

Differentiation of cells is most often assessed by semiquantitative or quantitative morphological methods such as determination of cell size, number of extensions over number of cells, and extent of neurite growth or neurite length [11]. Neurofilaments are dominant intermediate filament found in neuronal cells that provide specific support for developing neurite and maintaining neuronal caliber by formation of filamentous cross-bridge [14]. Another method to study neuronal differentiation is to investigate the formation of neurofilaments, the major structural components of neuron [18]. Regeneration of peroneal neurite can be qualitatively evaluated by immunofluorescence staining of neurofilaments. Anti-neurofilament is a useful immunocytochemical marker for axons [19, 20]. Neuronal cells were treated with aqueous extract at 20 *μ*g/mL (w/v) and incubated for 48 hr. Figures 4(a)–4(c) show immunofluorescene staining of PC-12 cells. After incubation with *L. rhinocerus* extracts obvious enhancement of neurite outgrowth with extension that double the length of cell body diameter can be observed.

Cold water extraction has been reported to yield lower amount of extract than hot water extraction but it is usually applied in traditional medicine preparation [21]. Most of Basidiomycetes mushrooms contain biologically active polysaccharides which are important for modern medicine [22]. Aqueous extracts contained high proportion of water-soluble constituents such as polysaccharides, such as *β*-glucan and water soluble components. Studies showed that aqueous extraction was employed to extract water-soluble polysaccharide from edible medicinal mushrooms. However, bioactive compounds from medicinal plants extracted with ethanol performed better activities than aqueous extractions [23] but aqueous extraction is believed to have lower cytotoxicity effect than ethanol extraction [24].

Wong et al. reported that freeze drying may be the best method for long-term preservation of bioactive compounds in mushroom responsible for neurite outgrowth stimulation activity. Inactivation of bioactive compound via oxidation could cause short cellular outgrowth of neurite and insufficient elongation to be scored as neurite [9]. Lower concentrations of aqueous extract may contain less active compounds and therefore insufficient for neuron to sense and instruct neuronal elongation process. Higher concentrations of extract could have toxic effect and eventually cause nerve cell damage. Based on the data collected, sclerotium of *L. rhinocerus *displayed NGF-like properties due to obvious morphological alteration shown by PC-12 cells. Further work on fractionation of *L. rhinocerus* extract that possesses NGF-like properties needs to be explored.

NGF is a signaling molecule that plays an important role in the differentiation and survival of peripheral sensory and sympathetic neurons [25]. When PC-12 cells were treated with NGF, G1 phase of Interphase was blocked and differentiation was initiated [26]. PC-12 experienced a dramatic phenotypic change when treated with NGF [10]. Percentage of neurite bearing cells of NGF-treated cells at optimum concentration of NGF increased as much as five times compared to non-NGF-treated cells. NGF activates extracellular signal-regulated kinase (ERK)—mitogen-activated protein (MAP) kinase pathway is responsible of neurotrophic responses in PC-12 cells [27].

Neurite outgrowth takes place in neuron (*in vivo* and *in vitro *differentiation). When neurosecretory cells, a type of neuron, were exposed to neurotrophins, peptides, and adhesion proteins, such as glycoproteins [28, 29], the activity took several hours to few days to perform differentiation [28]. Two major growth factor receptor downstream cascades involved in cell survival and neurite outgrowth are the phosphatidylinositol-3-kinase-Akt (PI3K-Akt) pathway and Ras-mitogen-activated protein kinase (Ras-MAPK) pathway. PI3K-Akt is vital in mediating neurotrophin-promoted cell survival whereas Ras-MAPK may be involved in mediating neurite-outgrowth [30]. Atwal et al. study showed that activation of TrkB-Shc site mediates neuronal survival and axonal outgrowth via PI3K-Akt and Ras-MAPK pathway [31]. EGF and NGF are a potent activator of ERK1/2 pathway in PC-12 cell line [31] and sustained activation of NGF is crucial for neurite outgrowth [32]. *Lignosus rhinocerus* extract that possessed NGF-like properties may be responsible for the activation able to activate Akt which is required in combination with activated MAPK cascade proteins for neurite outgrowth.

Neurofilament protein acts as a major component of the axonal cytoskeleton and was synthesized and assembled for axonal transport in cell body. After axon has grown and connected to its target cell, diameter of axon may increase as much as fivefold. Therefore, neurofilament proteins increased during NGF-induced PC-12 differentiation [32] and also conferring some degree of cellular protection rather than causing rapid cell death during cell death mechanism [33]. Besides that, morphological alteration cells observation under microscope, indirect immunofluorescence technique is an alternative to assess neuronal differentiation by neuronal marker on PC-12 cell line.

Additive effect occurs when there is a combination of two or more drugs with same therapeutic effect and the result is the sum of the drug's effects [34]. NGF action was enhanced by the aqueous extract at concentrations of 10, 20, 30, and 40 *μ*g/mL (w/v) whereas showed a weaker activity at combination with 50 *μ*g/mL (w/v). Combination of aqueous extract and NGF neurite showed outgrowth activity.

Researchers found that some compounds extracted from mushrooms, for instance, tricholomalides from *Tricholoma *sp. [35], termitomycesphins from *Termitomyces albuminosus *[36], and dictyoquinazol and dictyophorines from *Dictyophora indusiata* [37, 38], were active toward neurons. Lysophosphatidylethanolamine from *Grifola frondosa* was found to induce activation of MAP kinase cascade in PC-12 cells and resulted in neuronal differentiation and suppression of serum deprivation-induced apoptosis [39]. *Tremella fuciformis* crude aqueous extract contains polysaccharides, including glycosaminoglycan, promoted neuritogenesis, peripheral nerve regeneration, and muscle reinnervation following a sciatic nerve lesion [40–42]. Park et al. reported that exo-polysaccharides from the culture broth of *Hericium erinaceus* were shown to enhance the growth and neuronal differentiation of rat pheochromocytoma cells (PC-12) [43]. Nonpolar compounds of *H. erinaceus* known as erinacines extracted from mycelium have been well investigated and proven as strong stimulators of NGF [44, 45]. It promotes NGF gene expression *via* JNK signaling by inducing phosphorylation of JNK and its downstream substrate c-Jun and increased c-fos expression [46].

Water extracts of the rhizome of a traditional herb, *Coptis chinensis*, induced the growth of axon and dendrite and showed a sustained neurite outgrowth for a longer period whereas NGF-induced neurite did not maintain a fully differentiated state after 14 days in culture [47]. The nonpolar fraction of an Ayurvedic herb, *Centella asiatica*, known as asiatic acid showed significant neurite outgrowth activity on human SH-SY5Y cells [48].

Our results indicated that aqueous extract of *L. rhinocerus* contained compound/s that have potent NGF-addtive effect. It will be important to discover the compounds in *L. rhinocerus *that stimulate neurite outgrowth activity. As this mushroom is currently being investigated to validate traditional uses, the chemistry is not well known. Aqueous extracts are said to certain high molecular weight polysaccharides that have anti-inflammatory properties [49]. To our knowledge, this is the first report on neurite outgrowth activity of *L. rhinocerus. *With further development and investigation, *L. rhinocerus* and its bioactive compounds may be developed as mushroom nutraceutical to enhance process of neurite outgrowth activity in an aging population of humans.

Further detailed investigations are needed to ascertain clinical use of the extract in treating any neurodegenerative diseases and bioactive compounds that are responsible for neurite outgrowth activity have to be elucidated. This mushroom has been used as traditional medicine by indigenous folks of Malaysia for more than 15 diseases. The ethnopharmacological knowledge has to be scientifically validated to enable use of this mushroom as a nutraceutical or pharmaceutical. The findings warrant further studies of *L. rhinocerus* not only for nerve regeneration but also as a therapeutic agent for neurodegenerative diseases.

## 4. Conclusion

Aqueous extract of *L. rhinocerus* sclerotium contained NGF-like compound/s that enhanced neurite outgrowth activity. There was an enhanced stimulation of 17% of neurite outgrowth when a combination of 20 *μ*g/mL (w/v) aqueous extract and 30 ng/mL (w/v) NGF was added to PC-12 compared to the aqueous extract alone.
